# Identification of a 3-miRNA Signature Associated With the Prediction of Prognosis in Nasopharyngeal Carcinoma

**DOI:** 10.3389/fonc.2021.823603

**Published:** 2022-01-27

**Authors:** Jinhui Zhou, Bo Zhang, Xin Zhang, Chengyu Wang, Yu Xu

**Affiliations:** ^1^ Department of Otolaryngology Head and Neck Surgery, The Affiliated Huai’an No.1 People’s Hospital of Nanjing Medical University, Huai’an, China; ^2^ Teaching and Research Section of Otolaryngology, Hubei University of Science and Technology, Xianning, China

**Keywords:** nasopharyngeal carcinoma, WGCNA, miRNA-mNRA, lncRNA-mRNA co-expression network, prognostic marker

## Abstract

Nasopharyngeal carcinoma (NPC) is a malignant tumor caused by an infection of the epithelial cells of the nasopharynx, which is highly metastatic and aggressive. Due to the deep anatomical site and atypical early symptoms, the majority of NPC patients are diagnosed at terminal stages. There is growing evidence that microRNAs offer options for early detection, accurate diagnosis, and prediction of malignancy treatment response. Therefore, the purpose of this article was to identify microRNAs that predict the prognosis of patients with NPC by integrating biological information analysis. In this study, we utilized the GSE36682 dataset rooted in the Gene Expression Omnibus (GEO) data bank, including 62 cases of NPC tissues and six cases of non-cancerous tissues. The miRNAs were subjected to weighted gene co-expression network analysis, and hub miRNAs were screened for differentially upregulated miRNAs from modules highly correlated with tumor progression. We took a lot of time to calculate the risk scores of miRNA markers for 62 NPC patients, and incidentally combined the clinical survival information of patients to finally identify the three key miRNAs, and then divided the patients into low- and high-risk groups. Kaplan-Meier curve analysis revealed that the overall survival of patients in the high-risk group was obviously shorter than that of the low-risk group. Subsequently, the target genes of the three miRNAs were predicted and analyzed for functional enrichment. In summary, a prognostic predictive risk model based on three miRNA profiles may increase prognostic predictive value and provide reference information for the precise treatment of nasopharyngeal carcinoma.

## Introduction

Nasopharyngeal carcinoma (NPC) is one of the frequently occurring malignancies of the head and neck, with a unique geographic distribution and a relatively high incidence in Southeast Asia, including in southeastern China (as well as Guangdong and Hong Kong), India, and Thailand (1–3), though it is fairly rare in Western countries (4). It is generally accepted that the development of nasopharyngeal carcinoma is closely associated with the Epstein-Barr virus (EBV), cumulative inheritance in the context of susceptibility genetics and environmental factors, and genome-wide epigenetic modifications of tumor-associated genes (5). For the past few years, the mortality and incidence of nasopharyngeal carcinoma have gradually decreased due to effective screening and treatment strategies such as radiotherapy and combination chemotherapy, as well as the advent of immunotherapy (6–8). However, local recurrence and distant metastases are very common in patients with advanced NPC. Statistically, more than 30% of stage III and IV patients develop local recurrence or distant metastases within five years of receiving combination therapy (9, 10). Therefore, there is a need to clearly identify some sensitive biomarkers and new therapeutic targets for pre-NPC diagnosis and provide effective therapeutic measures.

The development of NPC is a complex, multistep, multifactorial, and aberrantly regulated process of signaling pathways involving aberrant activation of certain inactivation and oncogenes of tumor suppressor genes (11). Non-coding RNAs are usually dysregulated in tumor pathogenesis. MicroRNAs (miRNAs), a class of functionally short non-coding RNAs, are related to all markers of carcinoma development, metastasis, and progression, and have become one of the hot spots in carcinoma research (12). Elicited from microarray-based transcriptome profiling, an increasing number of studies have shown that many miRNAs are closely associated with the development and progression of nasopharyngeal carcinoma. For example, Jiang et al. (13) found that EBV-encoded miR-BART2-5p showed an elevated pattern in the serum of preclinical NPC patients and could promote NPC metastasis by inhibiting Rnd3. Zhou et al. (14) analyzed microarray data from the plasma of nasopharyngeal carcinoma patients and highlighted miR-548 and miR-940 as potential diagnostic biomarkers for nasopharyngeal carcinoma with high sensitivity and specificity. In addition, there have also been many studies conducted to find miRNA signatures associated with the prognosis of nasopharyngeal carcinoma *via* high-throughput miRNA expression profiling. Bruce et al. (15) identified a 4-miRNA signature (miR-140, miR-449b, miR-154, and miR-34c) associated with the risk of distant metastasis. Zhang et al. successfully identified a 4-miRNA signature by integrating biological information analysis for predicting the prognosis of patients with nasopharyngeal carcinoma. However, although many studies have analyzed miRNA expression profiles in nasopharyngeal carcinoma, unfortunately the detailed molecular mechanisms of NPC have not been fully elucidated.

In this research, we identified miRNA expression based on 62 nasopharyngeal carcinoma patient samples in the GSE36682 dataset, subjected them to weighted gene co-expression network analysis (WGCNA), and identified miRNAs associated with nasopharyngeal carcinoma progression. A prognostic prediction risk model was constructed based on the 3-miRNA signature to provide prognosis prediction, diagnosis, or precise treatment for potential candidate biomarkers of nasopharyngeal carcinoma.

## Methods

### Data Sources

We obtained research from the Gene Expression Omnibus database using the keywords “nasopharyngeal carcinoma” and “miRNA,” and then manually reviewed and selected cohorts containing miRNA expression and clinical survival information. The training GSE36682 dataset (platform: GPL15311, Human miRNA 1K) was obtained from Sun Yat-sen University, China and contained 62 nasopharyngeal cancer tissues and six non-cancerous tissues; the validation GSE32960 (16) dataset contained 312 nasopharyngeal cancer tissues and 18 normal tissues, with clinical survival data from the Zhang et al. (17) study obtained from the original investigators.

### Differential Expression Analysis

Differential expression of miRNAs was studied with the limma R software package; “adjusted P value<0.05 and |logFC|≥1” was defined as the threshold miRNA differential expression screen, and heatmap and volcano maps were plotted using the pheatmap and ggplot2 packages, respectively.

### Weighted Gene Co-Expression Network Analysis (WGCNA)

Using the WGCNA online resource, Pearson correlation coefficients between genes were calculated by using miRNAs from the WGCNA dataset and appropriate soft thresholds were selected β As a backup. A one-step method was used to construct a gene network, transforming the adjacency matrix into a topological overlap matrix (TOM) and generating a hierarchical clustering tree of genes. The DynamicTreeCut method was used to identify highly correlated co-expressed gene modules with the threshold set to cutHeight = 0.2 and minSize = 20. The Pearson correlation test was used to analyze the relationship between module signature genes (MEs) and clinical features.

### Lasso Regression Analysis

First, we performed the lasso regression algorithm, and 10-fold cross-validation was used to determine the parameters to obtain a suitable model. The multivariate Cox regression analysis was then performed on the genes obtained from the lasso regression, and the multivariate regression coefficient was calculated for each miRNA. Finally, the risk score equation could be constructed. Patients were divided into high- and low-risk groups according to the optimal risk score cutoff values. We first compared the overall survival time of the two groups, which would use to Kaplan Meier survival curve analysis, and subsequently evaluated the predictive value of the miRNA markers *via* time-dependent ROC.

### Cell Culture

Human nasopharyngeal epithelial cells NP69 and human nasopharyngeal carcinoma cell line SUNE-1 cells were rooted in the Shanghai Cell Bank, Chinese Academy of Sciences. SUNE-1 cells were cultured in RPMI1640 medium containing 10% fetal bovine serum (FBS) and NP69 cells were cultured in keratinocyte/serum-free (K-S) medium containing growth factors (Gibco, Grand Island, NY, USA). The serum-free (K-SFM) medium and all cells were cultured in a 37°C, 5% CO2 incubator.

### Fluorescence Quantitative PCR

Total RNA was obtained from NP69 and SUNE-1 cells according to the instructions of TRIzol (Invitrogen, USA). 1 μg of RNA was reverse transcribed into cDNA according to the instructions of the PrimeScriptTM RT reagent kit (TaKaRa, Japan) and 1 μg of RNA was reverse transcribed into cDNA according to the instructions of the SYBR Green reagent (TaKaRa, Japan). PCR amplification was performed according to the instructions of the SYBR Green reagent (TaKaRa, Japan). The PCR reaction system was as follows: 1 μL cDNA, 0.5 μL each upstream and downstream primers, 10μL SYBR Premix Ex Taq, 8μL ddH2O. The reaction conditions were: pre-denaturation at 95°C for 7 mins, followed by pre-denaturation at 95°C (5s), 60°C (30s), and 72°C (3 mins). The reaction was performed for 35 cycles at 95°C (5s), 60°C (30s), and 72°C (3 mins). The 2^-ΔΔCT^ method calculated the relative expression of target genes. The experiments were repeated three times and the mean values were taken. The primers used in this study were listed in **Table 1**.

**Table 1:** The sequence of the primers.

Subject	Primer sequence
ebv-miR-BART19-3p	Forward: 5’-CGCGTTTTGTTTGCTTGGG-3’ Reverse:5’-AGTGCAGGGTCCGAGGTATT-3’
hsa-miR-135b	Forward:5’-CGCGTATGGCTTTTCATTCCT-3’ Reverse:5’-GCGCGTAACACTGTCTGGTAA-3’
hsa-miR-141	Forward: 5’-AGTGCAGGGTCCGAGGTATT-3’ Reverse: 5’-AGTGCAGGGTCCGAGGTATT-3’
U6	Forward:5'-GACTGCGCAAGGATGACAC -3' Reverse:5'-CAGTGCGTGTCGTGGAGTC-3'

### Prediction of miRNA Target Genes

Three miRNAs (ebv-miR-BART19-3p, hsa-miR-135b, and hsa-miR-141) were analyzed using the online tool tarBase v8.0 for potential target genes. The target gene selection threshold was prediction score ≥ 8.0.

### Functional and Pathway Enrichment Analysis

Kyoto Encyclopedia and Gene Ontology (GO) of Genes and Genomes (KEGG) pathway enrichment analyses were performed on target genes using Data Bank for Integrated Discovery (DAVID) (v6.8) Visualization and Annotation.

### Statistical Analysis

Similar experiments were repeated at least three times in this study and expressed using the mean ± standard deviation. The data were subjected to an independent samples t-test and multi-factor ANOVA using Graphpad Prism 6.0.

## Results

### Identification of Differentially Expressed miRNAs

**Figure 1:**
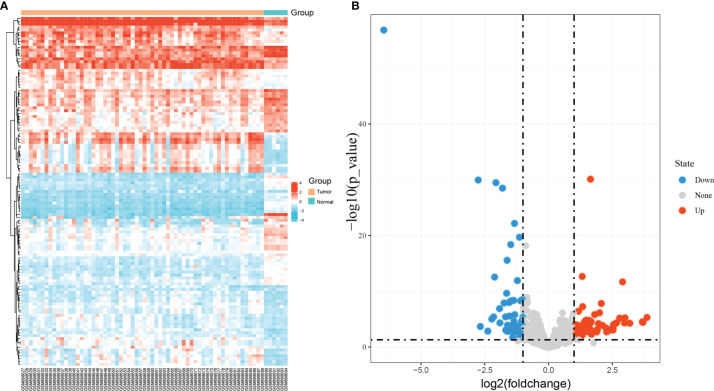
Identification of differentially expressed miRNAs in GSE36682. **(A)** heat map showing differentially expressed miRNAs; **(B)** volcano map showing differentially expressed miRNAs.

The GEO database was used to obtain the NPC-related miRNA expression dataset GSE36682, which included 62 NPC specimens and six non-cancerous tissue specimens. Next, a total of 121 DEmiRNAs were obtained using |logFC| ≥ 1 and adjust P < 0.05 as screening thresholds (**Figure 1A**). These included 63 expressing upregulated miRNAs and 58 expressing downregulated miRNAs (**Figure 1B**).

### Identification of Gene Co-Expression Modules

**Figure 2:**
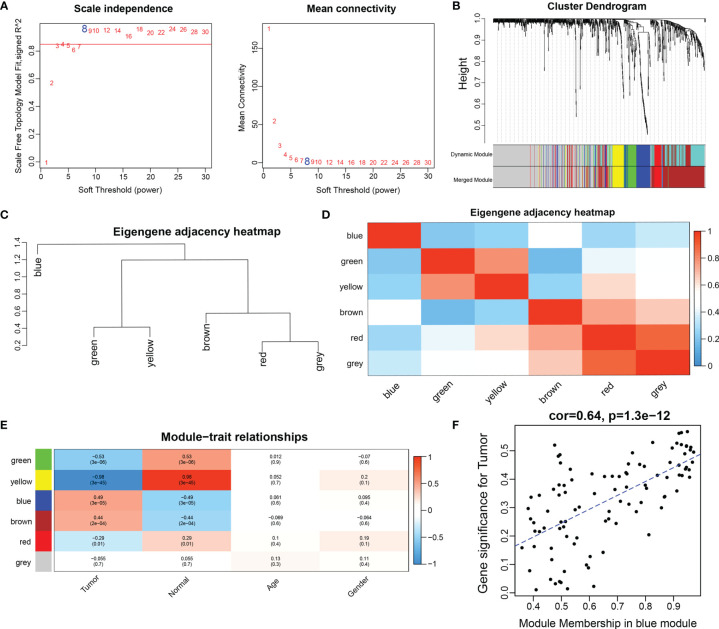
Construction of weighted gene co-expression network. **(A)** network topographies with different soft threshold powers; **(B)** gene tree diagram obtained by clustering based on the same topological overlap with the corresponding color rows indicating the module colors; **(C)** clustering analysis of each module; **(D)** correlation analysis of each module; **(E)** correlation between each module and clinical information; **(D)** scatter plot of correlation between gene module affiliation and gene significance in blue modules). **(F)** scatter plot of correlation between gene module affiliation and gene significance in blue modules.

To explore the miRNA expression patterns in NPC, we subjected miRNAs in GSE36682 to WGCNA analysis. To ensure a scale-free network, we chose a soft threshold of β = 8 (**Figure 2A**), generated a hierarchical clustering tree using the WGCNA package as the soft threshold power, and then identified a total of six modules (**Figures 2B** **–D**). Then, we constructed a co-expression network of associations between clinical features and these modules (**Figure 2E**). Notably, the blue modules were significantly positively correlated with tumor progression. Therefore, the blue module most associated with tumor development status was defined as the SUR module. The scatter plot of correlation between gene module affiliation and gene salience in the blue module is shown in **Figure 2F**.

### Hub miRNAs and the Survival Prognosis Prediction Model

**Figure 3:**
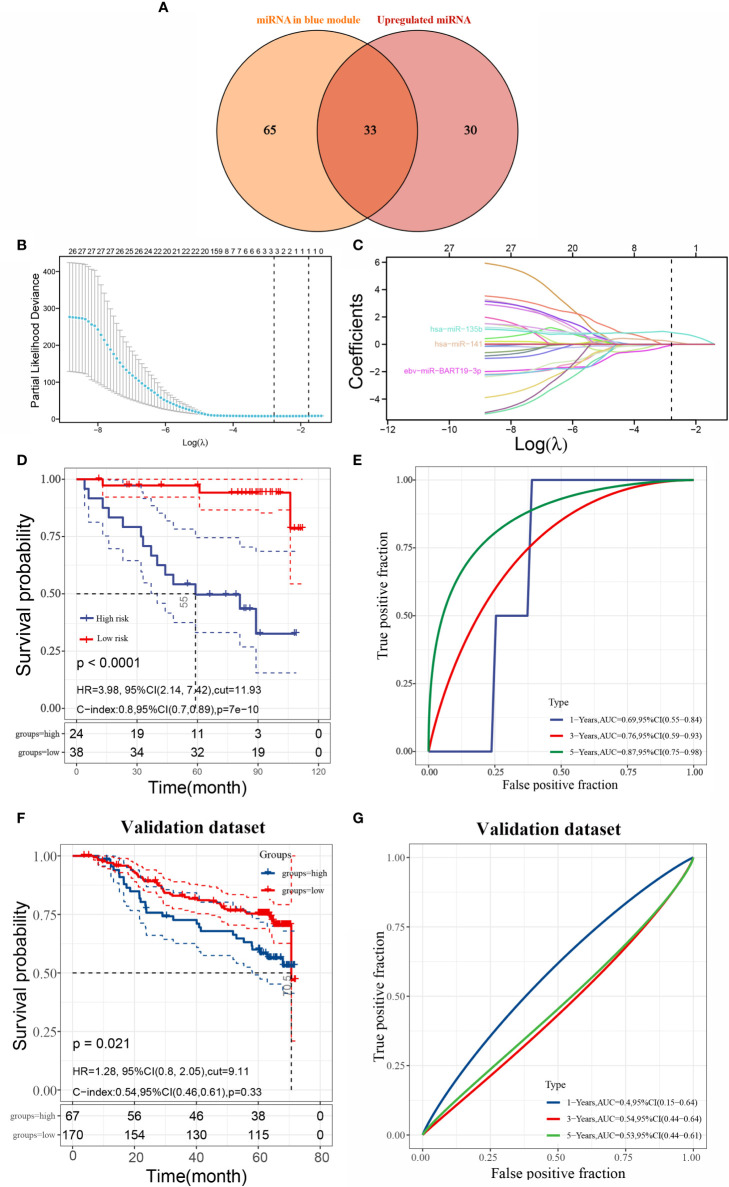
Identification of hub miRNAs and construction of survival prognosis prediction model **(A)** intersection of differentially upregulated miRNAs and miRNAs from the blue module; **(B)** lasso coefficient screening; **(C)** trajectory plot of lasso variables; **(D)** Kaplan-Meier curves of overall survival (OS) derived from risk scores of three miRNA features in the training set; **(E)** comparison of risk scores of three miRNA markers in the training set for predicting 1-, 3-, and 5-year ROC curves for survival; **(F)** Kaplan-Meier curves for overall survival (OS) derived from the risk scores of the three miRNA signatures in the validation set; **(E)** ROC curves comparing risk scores of three miRNA markers in the validation set for predicting 1-, 3-, and 5-year survival). **(G)** ROC curves comparing risk scores of three miRNA markers in the validation set for predicting 1-, 3-, and 5-year survival.

A total of 98 miRNAs were rooted in the blue module and overlapped with 63 variedly upregulated miRNAs, so that a total of 33 hub miRNAs could be obtained (as in **Figure 3A**). Next, we constructed a prognostic signature by using the lasso Cox regression model to analyze the expression levels of hub miRNAs. A predictive 3-miRNA signature model (**Figures 3B, C**) was constructed based on the minimal criterion (Lambda.min = 0.0614) selecting ebv-miR-BART19-3p, hsa-miR-135b, and hsa-miR-141, whose predictive risk scores consisted mainly of the following: Riskscore=(-0.05623)*ebv-miR-BART19-3p+(0.8671)*hsa-miR-135b+(0.1991)*hsa-miR-141.

The sample was divided into high- and low-risk groups according to the risk score ranking, using the best cut-off risk score as the threshold. The results of Kaplan-Meier analysis showed that patients in the high-risk group had a significantly worse prognosis than those in the low-risk group (**Figure 3D**). The model for predicting the overall survival (OS) period of patients was verified by applying ROC curves, and we found that the AUC values of this risk model for predicting prognosis at 1, 3, and 5 years were 0.69, 0.76, and 0.87, respectively, indicating that the model has high accuracy in predicting prognosis survival of patients with nasopharyngeal carcinoma (**Figure 3E**). In addition, we also validated the model using the validation set GSE32960, and the results showed that the prognosis of high-risk patients was significantly worse than that of low-risk patients in the validation set (**Figures 3F, G**).

### Hub miRNA Expression and Survival Prognosis

**Figure 4:**
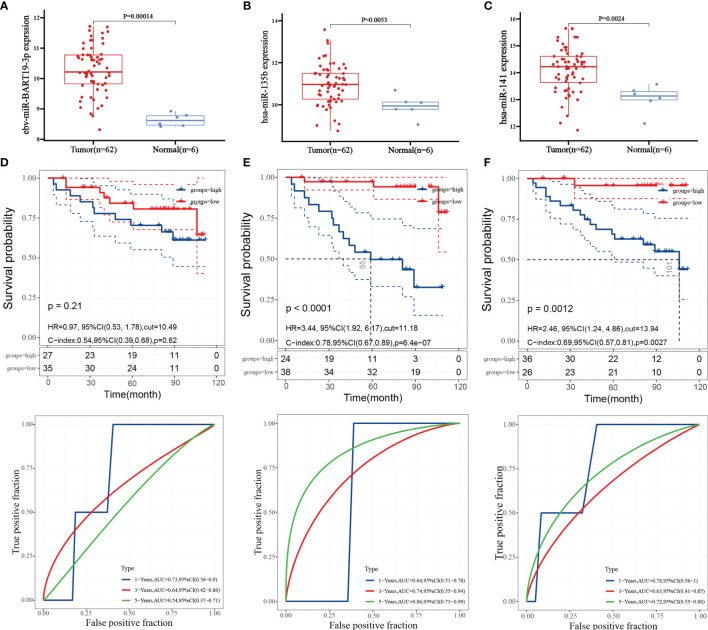
Hub miRNA expression and survival prognosis. **(A)** expression level of ebv-miR-BART19-3p; **(B)** expression level of hsa-miR-135b; **(C)** expression level of hsa-miR-141; **(D)** Kaplan-Meier survival curve and ROC curve of ebv-miR-BART19-3p; **(E)** Kaplan-Meier survival curve and ROC curve of hsa-miR-135b; **(F)** Kaplan-Meier survival curves and ROC curves for hsa-miR-141.

By analyzing the expression of three miRNAs in patients with nasopharyngeal carcinoma, the results showed that ebv-miR-BART19-3p, hsa-miR-135b, and hsa-miR-141 were upgraded in nasopharyngeal carcinoma tissues compared to normal tissues (**Figures 4A** **–C**). The patients were split into high- and low-expression groups, and it was found that the overall survival of those with high expression of hsa-miR-135b and hsa-miR-141 was significantly shorter than that of patients in the low expression group (**Figures 4E, F**), and the difference was significant (P < 0.05); however, the expression level of ebv-miR-BART19-3p was not associated with overall survival, with the difference not being significant (P > 0.05, **Figure 4D**).

### qRT-PCR

**Figure 5:**
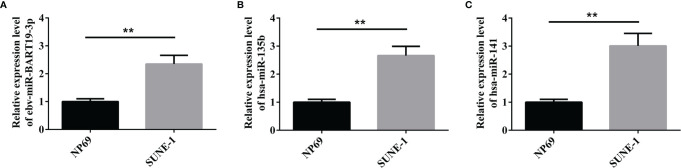
qPCR to verify the expression of the three miRNAs. **(A)** expression level of ebv-miR-BART19-3p; **(B)** expression level of hsa-miR-135b; **(C)** expression level of hsa-miR-141; **P < 0.01.

We detected the expression of ebv-miR-BART19-3p, hsa-miR-135b, and hsa-miR-141 in nasopharyngeal carcinoma cell lines *via* real-time fluorescent quantitative PCR, and the results showed that compared to human nasopharyngeal epithelial cells NP69, ebv-miR-BART19-3p, hsa-miR-135b, and hsa-miR-141 expression levels were significantly higher in human nasopharyngeal cancer SUNE-1 cells (P < 0.05, **Figure 5**).

### Predicted Target mRNAs

**Figure 6:**
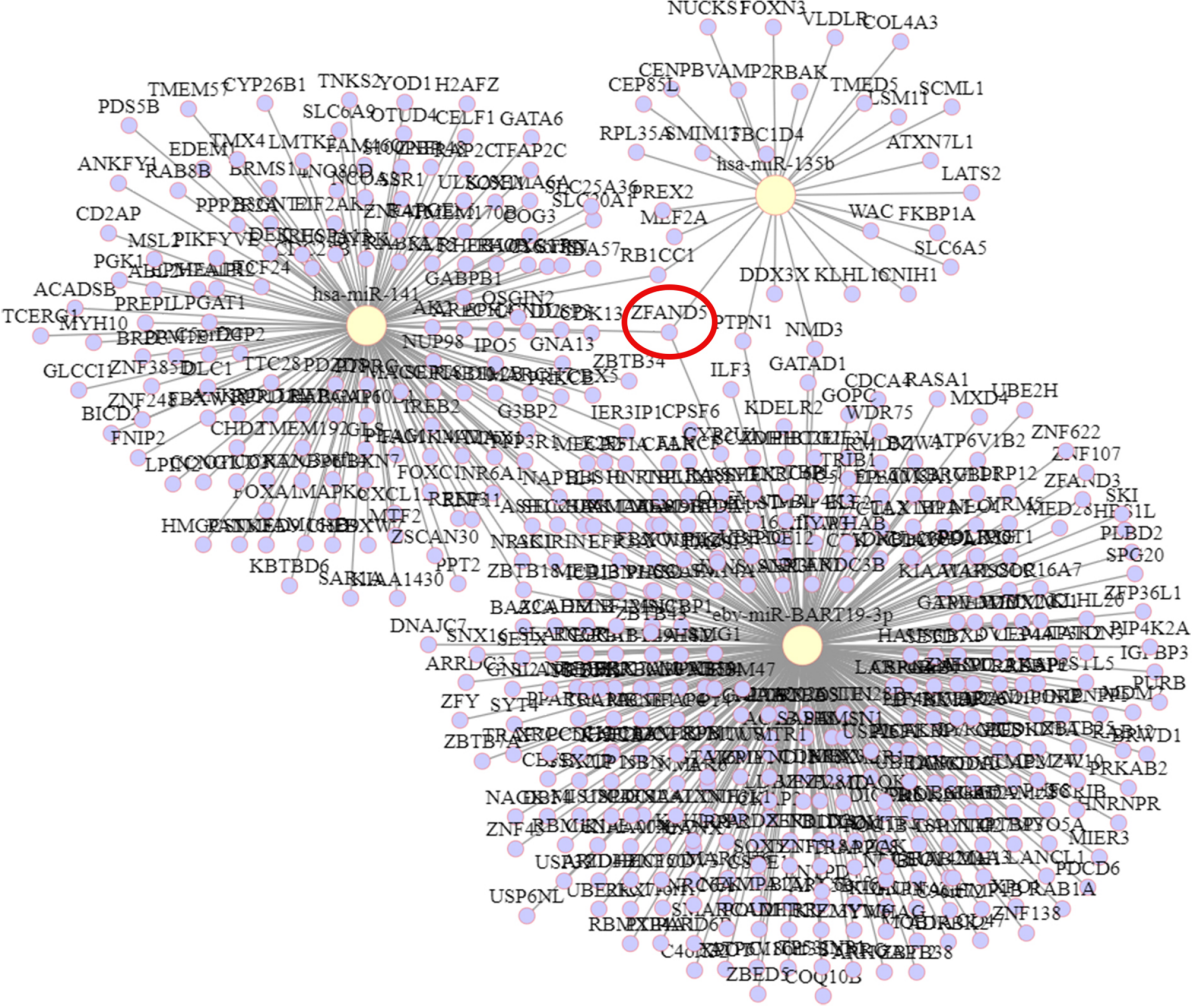
Predicted target mRNA of miRNA.

The target genes of hsa-miR-135b, ebv-miR-BART19-3p, and hsa-miR-141 were predicted by 335, 28, and tarBase v8.0, respectively, with 151 target genes obtained. The targeting relationships are shown in **Figure 6**. Among them, ZFAND5 could be observed as a common target gene for all three.

### Target Gene Function and Pathway Enrichment Analysis

**Figure 7:**
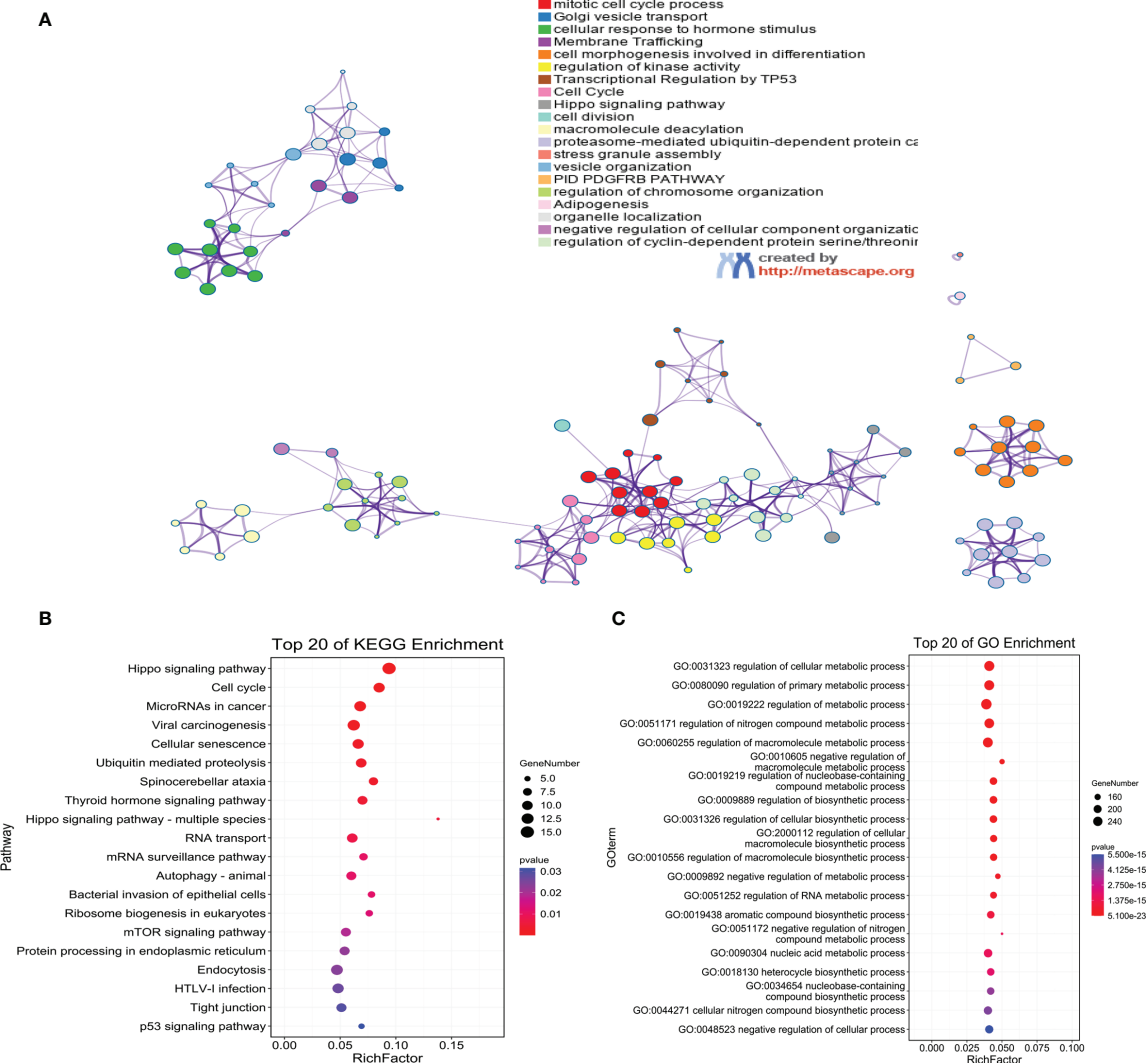
Functional annotation of target genes. **(A)** PPI network diagram of target genes; **(B)** KEGG enrichment analysis; **(C)** GO enrichment analysis.

The predicted target genes of ebv-miR-BART19-3p, hsa-miR-135b, and hsa-miR-141 were analyzed through the online analysis website Metascape to obtain PPI protein network interactions and visualize the gene information and network construction, as shown in **Figure 7A**. The KEGG and GO enrichment analyses of the predicted target genes are shown in **Figures 7B** **, C**. The results of the KEGG analysis revealed that the predicted target genes were mainly enriched in the Hippo signaling pathway, cell cycle, and MicroRNAs in cancer; the results of the GO analysis showed that the predicted target genes were mainly enriched in cellular metabolic processes.

## Discussion

In recent years, targeted therapies, less toxic and more effective chemotherapy modalities, and new technologies have provided new ideas for the treatment of nasopharyngeal carcinoma (18). Among them, gene therapy has received widespread attention for its increasing potential as a novel therapeutic approach (19). Beyond affecting all biological processes, including apoptosis and proliferation, miRNAs are unquestionably the best candidates for therapeutic agents. One of the most important advantages of miRNAs over conventional methods that target a single gene is their ability to target multiple molecules. This makes them very effective in regulating different biological processes related to normal and malignant cell biology (20). In turn, the most critical and significant challenge in the development of miRNA-based therapeutic modalities lies in identifying the best miRNA candidate or miRNA target for each disease. Therefore, the search for new key prognostic biomarkers is an urgent need if we wish to improve the prognosis and clinical treatment of patients with nasopharyngeal carcinoma.

In this research, the GSE36682 dataset was used to construct a co-expression network of clinical features and intermodular associations, i.e., to select modules related to NPC occurrence and development, and ultimately to identify 33 differentially upregulated hub miRNAs *via* the common method of differential expression analysis. Then, a prognostic prediction risk model based on the three miRNA signatures was constructed using lasso regression analysis, and the prognosis prediction of NPC patients at 1, 3, and 5 years was very precise. The three miRNAs, ebv-miR-BART19-3p, hsa-miR-135b, and hsa-miR-141, were highly expressed in nasopharyngeal cancer. hsa-miR-141 and hsa-miR-135b patients with high expression had a significantly worse prognosis than those with low expression, but ebv-miR-BART19-3p expression levels were not significantly correlated with prognosis.

The Epstein-Barr virus (EBV) is one of eight human herpes viruses that infect more than 95% of the world’s population (21). EBV persists as a latent infection in the B-lymphatic system throughout life and maintains a well-balanced relationship with humans. Once the fragile EBV-host equilibrium is disrupted, EBV can show its pathogenic potential (22). It is now well known that EBV belongs to one of the mechanisms of nasopharyngeal carcinogenesis, and some EBV genomic products, such as viral proteins, RNAs, and miRNAs, may be involved in nasopharyngeal carcinogenesis (23). EBV also expresses 44 mature microRNAs, including the BamHI fragment H rightward open reading frame 1 (BHRF1) miRNA and the BamHI A rightward transcript (BART) miRNAs (24). Among them, BART miRNA is highly expressed in nasopharyngeal carcinoma and EBV-associated gastric cancer and regulates the expression of multiple genes at the post-transcriptional level (25, 26). During NPC development, a minimum of 105 host genes are regulated by EBV miRNAs, which affect five important signaling pathways, including Wnt signaling (27). Zhang et al. (28) constructed the EBV-encoded miRNAs in chronic active EBV infection (CAEBV), EBV-associated phagocytic syndrome (EBV-HLH), and nasopharyngeal carcinoma (NPC). A comprehensive expression profile of EBV-encoded miRNAs in patients showed that miR-BART19-3p was upregulated in all these diseases and suggested that miR-BART19-3p is involved in the tumorigenesis of EBV-related diseases and may be a potential therapeutic target. In the present study, we demonstrated high expression of ebv-miR-BART19-3p in nasopharyngeal carcinoma *via* dataset analysis and fluorescent quantitative PCR analysis. has-miR-135b was first discovered to play a role in somatic stem cell differentiation (29, 30). hsa-miR-135b has been described in the literature as an oncogenic factor in most tumor tissues, such as colon cancer (31), oral cancer (32), and breast cancer (33), while hsa-miR-135b expression was upregulated in metastatic tissues of nasopharyngeal carcinoma (34). Similarly, the results of the present study demonstrated that hsa-miR-135b was highly expressed in nasopharyngeal carcinoma cells. miR-141 is a member of the miR-200 family (35). Dysregulation of miR-200 family members in cancer is associated with growth, apoptotic response, and metastasis regulation (36). However, numerous studies have yielded conflicting results regarding the role of miR-141 in the progression of different tumor types (37, 38), though some previous studies (39) have confirmed that miR-141 is upregulated in nasopharyngeal carcinoma tissues and that high miR-141 levels are negatively correlated with overall survival in nasopharyngeal carcinoma patients. In the present study, we also obtained the same results, where miR-141 was highly expressed in nasopharyngeal carcinoma cells and analysis of the dataset samples revealed that high miR-141 expression was significantly associated with poor prognosis in nasopharyngeal carcinoma patients.

In addition, miRNAs, as important regulators of biological pathways, can regulate target gene expression through translational repression or mRNA degradation and play a key role in tumorigenesis (40, 41). We subsequently predicted the target genes of ebv-miR-BART19-3p, hsa-miR-135b, and hsa-miR-141 and identified ZFAND5 as a common target gene for all three. ZFAND5, a member of the ZFAND family (42), enhances protein degradation by activating the ubiquitin-proteasome system (43). Finally, we performed KEGG and GO functional enrichment analyses of all target genes, and the results showed that genes were mainly enriched in the Hippo signaling pathway, cell cycle, and cellular metabolic processes. Initially, studies on the Hippo signaling pathway mainly focused on the regulation of organ size. Numerous studies have shown that the Hippo signaling pathway inhibits cell growth. In mammals, the upstream membrane protein receptors of the Hippo signaling pathway act as receptors for extracellular growth inhibitory signals and, once sensed, activate a series of kinase cascade phosphorylation reactions that ultimately phosphorylate the downstream effectors’ YAP and TAZ. Aberrant regulation of the Hippo pathway has been reported in several cases, including nasopharyngeal carcinoma (44, 45).

Of course, this study is not a perfect one, it still has some limitations. The sample size selected for the training dataset is too small, and we need a larger cohort to test the constructed miRNA signature to prove its robustness. Furthermore, while the prediction results and functional analysis for the target genes suggest that they are associated with some key signaling pathways, the functional analysis results need further experimental validation.

In summation, our work constructed a prognostic predictive risk model for patients with nasopharyngeal carcinoma based on three miRNA signatures (ebv-miR-BART19-3p, hsa-miR-135b, hsa-miR-141), which can be used to predict the overall survival of patients with nasopharyngeal carcinoma. In addition, the risk model combines human miRNA and EBV. This will provide new ideas for the development of novel therapeutic strategies for the diagnosis and treatment of nasopharyngeal carcinoma. However, experimental validation and detailed biological information analysis are needed for the miRNA downstream mechanism.

## Data Availability Statement

The original contributions presented in the study are included in the article/supplementary material. Further inquiries can be directed to the corresponding author.

## Author Contributions

All authors listed have made a substantial, direct, and intellectual contribution to the work, and approved it for publication.

## Conflict of Interest

The authors declare that the research was conducted in the absence of any commercial or financial relationships that could be construed as a potential conflict of interest.

## Publisher’s Note

All claims expressed in this article are solely those of the authors and do not necessarily represent those of their affiliated organizations, or those of the publisher, the editors and the reviewers. Any product that may be evaluated in this article, or claim that may be made by its manufacturer, is not guaranteed or endorsed by the publisher.
